# On Several Problems with the Application of P.Ya. Galperin’s Classical Theory

**DOI:** 10.11621/pir.2022.0402

**Published:** 2022-12-15

**Authors:** Andrey I. Podolskiy

**Affiliations:** a HSE University, Moscow, Russia

**Keywords:** Planned stage-by-stage formation of mental actions (PSFMA), System of Psychology, practical application of the theory of planned stage-by-stage formation of mental actions

## Abstract

**Background:**

The theoretical and applied works of Piotr Ya. Galperin have attracted the attention of scholars for more than 70 years. But what about the contemporary situation? Does the Galperin’s “System of Psychology” have only historical significance, or does it deal with crucial problems of contemporary psychology?

**Objective:**

This article explores several opportunities for applying Galperin’s System of Psychology and his theory of planned stage-by-stage formation of mental actions (PSFMA) as part of the System, in current conditions.

**Design:**

There are three main areas where the concepts of P.Ya. Galperin’s System of Psychology can be applied. The first is the application of the method of planned stage-by-stage formation to studying the formation and development of human mental activity. The second is the study of the theoretical and actual process of planned stage-by-stage formation as a psychological reality. The third area is the use of the provisions of the theory in the practice of teaching.

**Results:**

We argue that the efficacy of the provisions of the main components of Galperin’s System, and especially the PSFMA, is closely related to the solution of a number of purely theoretical issues today.

**Conclusion:**

The condition for the successful application of PSFMA principles is a harmonious combination of the basic psychological foundations of this process, taking into account the specifics of both the activity being formed, and of those socio-economic and technological parameters that create the space where such formation is carried out.

## Introduction

As we celebrate the 120th birthday of the outstanding Russian scientist P. Ya. Galperin, and pay tribute to his contributions to general, genetic, and educational psychology, it is necessary to highlight his method. His approach to the essence of mental phenomena and processes, and the mechanisms of their formation and development, was unique in its internal integrity and consistency. The doctrine of the subject of psychology, the objective necessity of the psyche, the main patterns of its development in phylo-, anthropo- and ontogenesis, and the patterns of formation of the elements of mental activity are the main components of Galperin’s psychological conception (Galperin, 2002).

The great heuristic potential contained in the works of P.Ya. Galperin is based primarily on the genuine internal integrity of his entire System of Psychology. Familiarity with any section of the System implies at least a general idea of the System as a whole. Unfortunately, the relatively small number of comprehensive publications written by Galperin during his lifetime, and his extremely concise writing style, have often led to the emergence of a number of misunderstandings and superficial interpretations over the more than 60-year history of this doctrine. Let us point out as an example the frequent confusion between the extended interpretation of Galperin’s theory (what we referred to as Galperin’s System of Psychology before), and the conception (theory) of the stage-by-stage (planned, planned stage-by-stage) formation of mental actions and concepts, which is, although crucial, only a part of this System, which significantly loses its status and heuristic potential if taken out of the context of the System as a whole (Arievitch & Haenen, 2003; Galperin, 2002; Podolskiy, 2010).

Today, there are at least three main areas where knowledge produced by P.Ya. Galperin’s System of Psychology can be applied.

The ***first*** is the use of the method of planned stage-by-stage formation to study the formation and development of human mental activity. (Arievitch, 2003; Hedegaard & Lompscher, 1999; Podolskiy, 2012; Stetsenko, 2017). The method of planned stage-by-stage formation (of course, to the extent that it can be implemented in accordance with the theory) becomes a touchstone by which various theoretical-psychological perceptions about the origin, structure, and functioning of various fragments of mental (primarily cognitive) activity can be tested for effectiveness and operation-alizability.

The ***second*** is the study of the theoretical and actual process of planned stage-by-stage formation as a psychological reality. (Galperin, 1969). Having singled out a number of areas in which the formation of specific mental actions takes place (transfer to an ideal plan, generalization, reduction, etc.), Galperin built a meaningful model of the functional-genetic process and indicated the main way to study it — the planned stage-by-stage formation of actions with specified indicators. At the same time, the discrepancy between the experimenter’s supposed and the actual course of the formation of the cognitive action was the stimulus to deepen the formulations by using the strategy of planned stage-by-stage formation.

Many years ago N.N. Nechaev (1975) developed what is figuratively called the tool, the use of which can allow the researcher to constantly control the nature of the relationship between the system of conditions which they have a priori described and constructed on the basis of the original theoretical model, and real experimental conditions; it is called “managing the process of controlled formation.” He added this most important point to the process of controlling the formation of cognitive actions which P.Ya. Galperin described. It is clear that this connection will be directly dependent on the completeness (always relative) of the initial model, and the depth of its reflection of the psychological mechanisms underlying the acquisition of its characteristics that are becoming cognitive actions. Considering that the features of the regulatory and controlling orientation of this action serve as such mechanisms, we can say that the path to a more complete study of the patterns of formation of cognitive actions goes through a comparison of the theoretical and real mechanisms of orientation of the formed cognitive activity, i.e., the hypothetical and real trigger conditions for these mechanisms.

And, finally, the ***third*** area for the application of the Galperin’s System of Psychology is the use of the provisions of the theory of planned stage-by-stage formation in the practice of teaching. Based on this theory and under his direct supervision, P.Ya. Galperin’s students and followers carried out several hundred projects aimed at improving the content, forms, and methods of education on all levels. These included preschool, primary school, secondary general education and vocational schools, higher education, training workers and specialists in production, advanced training and retraining of managers and specialists from various sectors of the national economy, and military and sports training.^1^ Over the last 15–20 years, there has been a significant expansion of Galperin’s approach in new areas, applying it to evaluation the moral competence of children and adolescents (Brugman et al., 2001), promotion of the psychological well-being of people at different stages of ontogenesis (Idobaeva, 2011), the development of professional consciousness (Nechaev, 2014), and other areas.

The main result of the theory’s application to the aforementioned areas was the following: learning time was reduced, while the quality of acquiring the relevant material was improved; successful learning for the vast majority of students was ensured; a significant increase in their interest in learning was observed; and differentiated learning while maintaining a single structure of theoretical knowledge became possible.

## Results

The more than 60-year history of Galperin’s approach gives every reason to assert that its competent use allows us to quite successfully solve many practical problems, providing both researchers and practitioners with a most powerful “intellectual tool” (Podolskiy, 1997; Engeness, I. & Edwards A., 2017). We will focus precisely on this area in this article. Although we will do this not only (and, perhaps, not so much) because the modern trends in the socio-economic life of society — “digitalization” of the economy, the need for training, or rather retraining (sometimes multiple times) throughout life, requirements ensuring the formation of the so-called “Competences of the 21st century” among the vast masses of the population, etc. — with all their acuteness put forward new challenges to the Galperin’s System (Seel, N. et al., 2017). Another reason for such an emphasis, which is more significant in our opinion, is that successful practical (truly practical!) training — especially in its mass version — requires, paradoxical as it may sound, notions about the mechanisms of formation of human mental activity that are much deeper and more extensive than arbitrarily “pure” academic laboratory research.

The key question that arises in this regard is: what is the reason for the relatively limited application of Galperin’s theory in the practice of training? It is this question that prompts the ongoing debate in national and international psychology and pedagogy about the real possibilities of using the conception in training practice (Engeness, I. & Lund, A., 2020; Podolskiy, 2020; Talyzina, 2020; Reshetova,1989). From our point of view, the key to solving this issue is a correct understanding of the scientific status of the conception itself.

Despite the outward “similarity” of the core of the conception — the formation of mental actions and concepts — to the main goal of almost any training, the theory of planned stage-by-stage formation of mental actions itself is not and never was a theory of training. The works of P.Ya. Galperin’s students and followers (N.F. Talyzina, 2020; Z.A. Reshetova, 1989; N.N. Nechaev, 2014; A.I. Podolskiy, 2010) and others) describe the additional, intermediate work that should be carried out by psychologists and teachers in order to move from the general psychological knowledge contained in Galperin’s basic conception, to the construction of the actual learning process — the actual content carried out in the interaction of a real teacher and real students.

Moreover, as has been repeatedly shown in the experiments of the last decade, fragments of the planned stage-by-stage formation procedure are not absolute and, in this sense, are external to the subject. They receive their psychological certainty only in a specific situation. The main condition for the effective practical application of the conception is not the aspiration to literally reproduce some abstract general procedure, but to have a creative psychological modeling of a specific situation (learning, information retrieval, interaction, etc.). If this requirement is met, the practical application of this approach indeed provides excellent results, as has been repeatedly demonstrated in relation to various components of school and university vocational training. Otherwise, if one tries to implement the theory in teaching as if it were a kind of universal knowledge, almost a philosopher’s stone, the result will as a rule be deplorable.

There is an interesting paradox: the more universal knowledge is (and Galperin’s System, from our point of view, contains highly universal knowledge), the more specific must be the auxiliary means by which this universal knowledge can be applied to specific cases. The less universal the knowledge, the less such specifications are required, because such knowledge is specific in itself. Indeed, in a general sense, it can be said that a person’s mastery of any knowledge, skill, or any new competence always presupposes some more or less complete orientation to the task, in the sense of what is happening, in the specific circumstances and conditions for achieving the accepted goal. In this respect notions formulated by P.Ya. Galperin about the structure of the orienting basis of action and the ways of its formation are indeed universal (Galperin, 2002; Engeness & Lund, 2018).

However, the way the process of forming an orientation to a particular subject area should be designed is another matter. It must be adjusted for the age-psychological characteristics of the students, taking into account the individual characteristics of quite specific children, adolescents, and adults, as well as individual psychological characteristics and pedagogical capabilities of the teacher, university professor, or vocational training instructor. How should one move from the most generalized scientific theoretical knowledge to technological, methodological knowledge, which, in fact, is required to be used in practice? Or, in other words, what prevents wide and full-fledged practical implementation of Galperin’s approach in practice?

Certainly, almost in the first place, the inhibitory factors are the same as those for the introduction of other psychological developments. However, there are a number of circumstances specific to Galperin’s conception. The first circumstance is rather external to the substantive side of the case. There is a huge distance between the non-psychologist’s preconceived notion of the simplicity of obtaining spectacular results with the help of PSFMA (theory of planned stage-by-stage formation of mental actions) tools and the true complexity of this process, which involves long painstaking work both in designing and in implementing training. Awareness of the need for a thorough subject analysis, building a psychological model of the forming activity, and the formation process itself, its specification in relation to the features of the material, the age and individual characteristics of the trainees, and many other components that make up the procedure of planned stage-by-stage formation, often, as our experience shows, turn out to be serious, if not decisive, obstacles in the way of the introduction of PSFMA.

More meaningful and, therefore deserving of serious analysis, are the circumstances related to the assessment of specialists in general, university, vocational, and other education, regarding the practical possibilities of PSFMA. Assertions about the limitations of these possibilities are not uncommon. As justification, the following reasons are given: 1) The use of PSFMA is associated with a radical breakdown of established methodological and organizational forms, which gives rise to practically insurmountable difficulties of an objective and subjective order; 2) The possibilities of PSFMA are limited by the formation of separate fairly simple actions, and since almost any activity in modern production is a complex hierarchy in which an action is only a particular element, is it worth spending effort, time, and money to take this step (even assuming that it produces results of a somewhat higher quality) without affecting the rest of the structure of professional activity; and 3) The application of the provisions of the PSFMA allows for the formation of only high-quality skills, while a student’s broader creative activity, the share of which in modern production is increasing, remains unaffected. Some other critical considerations are also given, but these are perhaps the main ones.

These considerations cannot be instantly dismissed by simply pointing out that their authors misunderstand the true meaning of the conception. The true task of a psychological researcher interested in the practical implementation of the scientific achievements of the PSFMA should be an objective analysis of the current situation and explanation to practitioners of the ways of applying the provisions of the conception, taking into account the current and future requirements of professional training.

Many difficulties in the practical implementation of the provisions of the PSFMA today are closely related to the solution of a number of purely theoretical issues. Historically, impressive pedagogical results of applying the theory of the controlled formation of action came to the fore in a significant part of the psychological research carried out in line with the conception of P.Ya. Galperin. Interest in truly extraordinary (in comparison with traditional methods) indicators of assimilation led in a certain period to the extensive development of research and the application of methods of planned formation to an ever-wider range of human actions. The reverse side of this generally positive process was the fairly common belittling of the status of the conception, relegating it to the level of a kind of methodological guideline, a practical manual regarding the organization of training.

If we recall the history of the emergence of Galperin’s theory and the first stages of its development, it is clear that initially it was about discovering the conditions for the formation of a separate action on a certain subject in a person’s mental plan. After these conditions were identified, it became possible to construct a generalized scheme of its stage-by-stage formation (later this term was replaced by the author with “planned” and “planned stage-by-stage”). Later, the main efforts of P.Ya. Galperin were aimed at clarifying the composition of these conditions, their systematization, and the application of the system to the formation of various types of cognitive actions and images. At the same time, the formation itself, no matter how detailed its development, still remained a means of implementing the research strategy, subordinated to the goals and objectives of a psychological research project. This presupposed, first of all, the acceptance of certain abstractions necessary for any scientific research. In this case, such abstractions were the assumptions about the isolated formation of a separate mental action, about the novelty of the formed action for the subject, the limitation of the ongoing psychological transformations to the zone controlled by the experimenter, etc.

## Discussion

It is easy to understand that the direct use of research methods in practical training has a number of fundamental limitations. Highly successful results in practical training cannot be expected to be maintained if circumstances which were deliberately put aside in a psychological experiment, come to the fore in a real training situation. This is precisely what happens most often. Hence the explicable discrepancy between the possibilities of experimental techniques and the real efficiency of their practical application. Such a direct transfer gives satisfactory results only in those cases, which are not too frequent, when the psychological content of the object of experimental formation completely (or, at least, mainly) exhausts the corresponding content of the object of real learning, and the other aspects of the latter are organized in such a way that they do not have a practical effect on this content.

We need to consider the fact that in order to fill the gap between research methods and training practice, it is necessary to carry out a whole range of developments involving a number of stages of the reality of training. It can be said that we need a special science that raises such an approach from the empirical level to the theoretical one. It is the needs of the practical use of the method of planned formation that lead to the need for additional reflection of its essential characteristics, and the separation of the internal patterns of acquiring a new activity from the specific forms of this acquisition described in numerous studies by the followers of P.Ya. Galperin. Unfortunately, these studies often confuse two different languages: 1) the language of the *conditions* for organizing orientation and execution of an action, and 2) the language of the *mechanisms* for the formation of orientation structures, the subject’s acceptance of these conditions, and their active reflection. This is another source of misunderstanding the true possibilities of the method by practitioners.

Focusing on an external procedure, reproducing it according to a certain general template without taking into account specific circumstances, and missing significant points or distorting their meaning — this is an incomplete list of the defects common to the practical application of planned formation methods. In our opinion, this is not the fault, but the misfortune of practitioners. Given all the attractiveness and seeming simplicity of obtaining effective results, behind each of them there is a thorough psychological and pedagogical process, which, unfortunately, has not yet been fully brought to the level of a technology

Meanwhile, P.Ya. Galperin strongly emphasized the need to distinguish between the external form of the method, which depends on the conditions of its application, and its actual content. The main and constant content of the method is the **set of steps** that must be carried out in order to obtain an action, representation, or concept with the desired given properties as a result of formation (Galperin, 2002). It should be noted that the concept of a set of steps includes not so much a description of external conditions as a description of the content and form of the subject’s own activity, its controlled changes through the creation of a controlled system of conditions. Then it becomes necessary to carefully analyze the psychological content of both the activity planned for formation and the process of formation itself.

Features of the tasks being worked on, the specifics of the activity (both in terms of its content and in terms of its place in the overall structure of production), age-psychological characteristics of the perception of the training situation, and other factors most significantly affect the layout of the procedure. In a number of cases, such familiar attributes of planned formation as the sequence of stages and the method of setting and assimilating the scheme of the complete orienting basis of the action, may vary. For example, for some professional activities it is not at all necessary to achieve a mental (ideal) form of performing their main components. Moreover, there are a number of professions in which regulations require the use of external support. There, the procedure for planned formation also takes on a form that is very far from the paradigmatic one. The same can be said about cases of retraining and mastering related professions, in which many of the psychological steps which P.Ya. Galperin describes have already been mastered by trainees earlier, and the point is not the formation of new psychological structures, but their actualization.

Finally, in the overwhelming majority of cases, practical (primarily vocational) training involves the formation of not one action, nor even a simple series of actions, but a most complex hierarchized system of actions. The development of even a laboratory version of the scheme for the formation of such a system is a rather difficult task.

From what has been said, it is clear that the productive application of the principles of planned formation presupposes carrying out diverse steps regarding the optimal “docking” of psychological requirements and specific features of the training situation. Skipping these steps and ignoring the psychological mechanisms behind them, leads to emasculation of the conception and a decrease in its practical potential.

Thus, the direct application of the procedure of planned formation in the practice of training should be preceded by a preparatory period. Primary and definitive here is the psychological analysis of the activity planned for acquisition, the structure of the process of its formation, and the training situation itself. The result of such an analysis should be the construction of *a psychological model* of a specific case of vocational training (Podolskiy, 2008). We, following P.Ya. Galperin, presuppose a quite definite understanding of the concept of the psychological. It is used to describe the patterns of formation, development, and functioning of the active orientation of the subject in a training situation; the content of the forms of orientation necessary for the implementation of a full-fledged professional activity in all its parts; the actions and images necessary to reveal this content at different stages of assimilation; the content of these stages; and special characteristics (for example, the objectively or subjectively intense nature of the professional activity itself or the process of mastering it) (Galperin, 2002).

To date, the PSFMA has accumulated a lot of data on the patterns of description and presentation of the content of the elements of human activity — individual actions and images, and the ways of their purposeful, controlled formation. However, no less important is the disclosure of the principle by which individual actions and images are formed into an integral structure of real activity. We place special emphasis on this point, since any modern, primarily professional activity is a more or less complex hierarchically organized system of actions that are diverse not only in their subject matter, but also in their psychological content, *i.e.,* the place occupied by these actions in the hierarchical structure of a person’s orientation in those relationships, circumstances, and characteristics that are essential for mastering and implementing high-quality professional activity.

It seems to us that the development of classifications of the types of such hierarchical models for the main groups of professions should be undertaken in the near future, which would greatly simplify the process of introducing the achievements of the PSFMA into practice. To date, we have several samples of the implementation of such contributions in constructing optimization options for training a number of workers and professionals. For example, the hierarchical structure of orientation was most notably presented in our development of the psychological foundations for the training of operators of nuclear power plants (Podolskij, 2010).

The next step would be to develop a system of conditions that ensured the construction of this complex activity. As is known, P.Ya. Galperin identifies a number of subsystems of psychological conditions that ensure the formation of activities with specified indicators (Galperin, 2002; Engeness & Lund, 2018). Since the orientation of each of the levels is a very complex activity with its own structure, it seems appropriate to build three relatively autonomous groups of these conditions for each level: 1) the formation of appropriate orientation bases; 2) the preparation of training and control tasks; and 3) the organization of controlled acquisition. At the same stage of development, indicators for the formation of individual components of the orientation at each level are outlined, and the development of specific methods for the formation of these components is planned; *i.e.,* along with the development of a general macro-scheme of formation, the necessary number of micro-plans of a kind is outlined, which are aimed at formation of individual actions and images.

An important point which must be taken into account at this stage is the age-psychological characteristics of the trainees. Ignoring these features (especially in relation to adolescence and early maturity) can lead to the trainees’ psychological devaluation of vocational training. To prevent this from happening, training should not only form high-quality knowledge and skills, but also ensure a conscious identification of a real connection between the characteristics of an activity (reflecting the success of the training that precedes it) and the parameters of its social and personal significance (economic, social, socio-psychological, moral).

Taking into account the age-psychological aspect is also significant for older ages. For example, the current widespread method of vocational training in the workplace, as schools for the dissemination of advanced techniques and methods of work, is not very effective. This is largely due to the fact that the “frontal” psychological procedure implies the insufficient professional competence of the participants, which, in turn, evokes various forms of psychological defense mechanisms in workers with a long job tenure. What is natural and positive for children — the counter-position of the bearer of knowledge (trainer) and the person lacking knowledge (trainee) — is not suitable for people at the age when they are especially sensitive to the assessment of their capabilities, in particular professional ones.

The solution is to change the procedure for organizing training schools: setting a certain standard, but encouraging all school participants to conduct a joint analysis, which leads to the identification of features of the activities of a successful specialist. In this case, all participants subjectively become, as it were, “co-authors” of the technique found. There is a rearrangement of psychological emphasis: not “they are better, I am worse,” but “we found it together; why are they doing it better?” In this approach, only one element of planned formation is subjected to age-psychological concretization — the objective conditions for the successful performance of the action for the subject.

It is easy to show that each component of the system of planned formation can’t be considered in isolation from the laws of human ontogenetic development, but, on the contrary, must be seen through the prism of such development. It is clear that this circumstance significantly affects the construction of a psychological model. Thus, the psychological model of training must include a description of the macro- and microstructure of orientation. However, this model is by no means a model of real learning; at the moment we are just talking about its framework.

The next stage is the construction of a ***psychological pedagogical model*.** Its main function is the projection of the psychological model on specific conditions: the organizational forms of the training, available technical training tools, its desired dynamics, etc.

The application of the conception of planned formation to the practice of training can pursue two goals: either the development of a new optimal training option, or the modernization of existing training methods. In the second case, the psychological pedagogical model will be built selectively, prescribing a partial revision of the content or form of training, and retaining empirically based successful moments. Bearing in mind that each phase of the existing training in this case will be considered through the prism of the psychological pedagogical task at which it is directed, advantageous opportunities open up for the purposeful use of pedagogical experience accumulated in the field.

The construction of a psychological pedagogical model ensures the imposition of a psychological model on selected *forms* of training, taking into account the specific situation. In this way the general form of the training process, and the sequence and content of its main fragments, are determined. The construction of a psychological pedagogical model creates real opportunities for extrapolating the practical effect of introducing a planned formation procedure.

The final stage of development is the construction of a ***methodological (technological) training model*.** This model includes a detailed description of the course of training; it fixes the place and time of each lesson, and establishes the success criteria for both each lesson and the course of study as a whole.

Even a brief description of these models indicates the complexity of the full-fledged, evidence-based implementation of the provisions of the PSFMA in training. However, the results obtained indeed show a meaningful return on investment. Furthermore, taking into account the acuteness of the issue of improving personnel in the modern economy, it can be confidently stated that the theory of planned formation in its modern form is one of the serious ways of solving this problem, operationally and long-term.

At the same time, the key to success is a deep, meaningful approach to both planning and ensuring implementation, an approach that no longer considers planned formation a purely scientific abstraction, but a reality that is influenced not only by the initial theoretical premises, but by the whole complex set of economic, production-related, and socio-psychological circumstances that determine the course and content of training specialists in production.

To date, the conception of planned formation has accumulated a lot of data on the methods of describing and presenting subject content in the purposeful formation of individual actions and images. However, any real human, not to mention professional, activity is a complex hierarchy of actions, diverse not only in its subject matter, but primarily in its psychological nature and place in the structure of a person’s orientation in those relations and circumstances that are essential for mastering and best performing any actual activity.

## Conclusion

In creating a theory and developing a method of planned formation, P.Ya. Galperin quite justifiably had to put aside a number of psychological characteristics of human activity. This allowed him to create a coherent theoretical structure and to work out the most effective method of psychological research on the formation of mental (more broadly, cognitive) activity. At present, from the theoretical, experimental, and applied sides, there is a need to take the next step in the development of scientific thought: to construct a psychological model of the process of formation of a specific cognitive action, which would include a description of the reflection by the subject on the most complete set of both subject-object and subject-subject relations implicitly contained in the situation of planned formation. The more complete and multi-layered the perceptions that are included in this model, the more intensive and productive our movement from potential to real explanatory and practical possibilities of the method of planned formation will be.

This is exactly what is demanded by the current challenges of the 21st century. National and international scientists and practitioners continue to carry out work in a number of areas. There are special requirements for human mental activity, the formation of which involves an increasingly large-scale deployment of the digital economy (Engeness & Morch, 2016), and a new stage of large-scale deployment of the Instructional Design movement, which has by no means lost its relevance, and quite favorably reacted to the connection of the Galperin direction to its developments (Seel, N. et al., 2017). An interesting challenge is to use the fundamental and concrete possibilities of Galperin’s approach to such a movement as the Partnership for 21st Century Skills (Partnership…, 2018). Laboratory research conducted by us and our colleagues becomes achievable when the psychological model of this process includes ideas about the genesis of the *multicomponent structure of the orientation* of the emerging cognitive action (Podolskij, 1997; 2010 ).

Here work needs to be done on all the levels of the theory, including in relation to phenomena that have not previously been analyzed in terms of planned formation. Thus, we deem it productive to consider the category of “properties (parameters) of an action,” not only as some characteristic of the objective state of a given action, but also from the point of view of their representation in the mind of the subject.

For example, the “level of action” parameter is not only an indication of how (with or without support from external means or speech) an action can be carried out. It inherently contains an indication of the social significance of this property: an action performed with the support of a given plan and means from outside will have a social valuation that differs sharply from the assessment of an action with the same content, but performed without such external mediation. Certainly, the “internal-external” (“material/materialized-ideal”) axis is not identical to the opposition of “bad-good.” In a number of cases, it is not the ideal, but the materialized implementation of an action (for example, the performance of regulated professional actions in an extreme situation) that will have a higher social significance.

Other properties of action, both primary and secondary, can be considered in a similar way. It is clear how much more complex the psychological model should become, taking into account not only the purposeful movement of the emerging action towards the intended indicators, but also the person’s subjective refraction of both these indicators in their social and personal meaning, and the functional genetic process itself.

In the future, a detailed analysis of this type can be carried out in relation to all components of the systems of planned formation. Thus, the subsystem of the formation of a complete orienting basis of an action requires consideration of the entire hierarchical structure of orientation; the internalization subsystem (transferring an action into an ideal plan), requires consideration of nonlinear changes in psychologically heterogeneous, albeit interconnected components of the structure of action regulation. The trend towards the convergence of the subjective and objective characteristics of the human psyche, and its motivational and operational components, is very typical for many areas of modern psychology (in particular, for the Partnership for 21st Century Skills movement). However, a truly meaningful solution to this issue can only be achieved on a clear methodological basis. “That which was separated from the very beginning cannot further be connected, except in an external way, but the bare declaration of their unity *(as in cognition and relation, objective and subjective content — A.P.)*, like any bare declaration in general, does not in fact change anything” (Leontiev, 1975, pp. 284–285).

In other words, the general methodological requirements for a holistic, systematic consideration of the aforementioned aspects of human mental activity have been outlined. Now it is up to specific theoretical and experimental developments to truly show the psychological integrity of the genesis of the components of human cognition, and the contradictory unity of its objective and subjective content, which we deeply believe is especially relevant in our stormy and incredibly dynamic times.

* * *

In the present publication, we dwelled only on some general issues of connection between the fundamental principles of the System of Psychology of P.Ya. Galperin and the possibilities of their application in 21st century practice. It is crucial to understand that the condition for the successful application of these principles is a harmonious combination of the basic psychological foundations of this process, taking into account the specifics of both the activity being formed, and those socioeconomic and technological parameters that create the space where such formation is carried out.

## Footnotes

1 In 1999, P.Ya. Galperin (posthumously) and a group of his students and followers were awarded the Russian Federation President Award in the field of education for the development and practical implementation of the provisions of this theory.
